# Investigation of the Visual Acuity Test Success Rate of a New Child-Friendly Minimum-Separable Chart for 2- and 3-Year-Old Children

**DOI:** 10.3390/vision9040100

**Published:** 2025-12-17

**Authors:** Yo Iwata

**Affiliations:** Department of Rehabilitation, Orthoptics and Visual Science Course, School of Allied Health Sciences, Kitasato University, Sagamihara 252-0373, Japan; iwatayo@kitasato-u.ac.jp; Tel.: +81-42-778-9667

**Keywords:** visual acuity, child-friendly chart, cooperation rate, pediatric ophthalmology, Landolt ring, early vision screening, toddler visual assessment

## Abstract

**Background/Objectives:** Early detection and timely treatment of amblyopia require reliable visual acuity testing in toddlers; however, conventional Landolt ring charts often show poor testability in 2–3-year-old children. Therefore, we aimed to verify the practicality of a new Child-Friendly Minimum-Separable (CFMS) chart for 2–3-year-old children by comparing cooperation rates with the standard Landolt ring visual acuity chart. **Methods:** A prospective pilot study was conducted on 20 children aged 2 years (30.6 ± 3.2 months) and 20 children aged 3 years (41.8 ± 3.9 months). Right-eye visual acuity was tested in random order using the Landolt ring (four options, 3/5 considered correct) and the CFMS chart (two options, 3/3 considered correct). Test cooperation rates and visual acuity were analyzed. **Results:** In the 2-year-olds, the cooperation rate was 15% and 75% for the Landolt ring and CFMS chart, respectively (*p* = 0.0005). Twelve children refused to cooperate with the Landolt ring but cooperated with the CFMS chart; the reverse did not occur. In the 3-year-olds, the cooperation rate was 60% and 90% for the Landolt ring and CFMS chart, respectively (*p* = 0.031); six children cooperated only with the CFMS chart. The odds ratio for cooperation per additional month of age was 1.34 (95% confidence interval [CI]: 1.12–1.59; *p* = 0.001) and 1.24 (95% CI: 1.03–1.50; *p* = 0.026) for the Landolt ring and CFMS chart, respectively. **Conclusions:** Compared to the Landolt ring, the CFMS chart significantly improves cooperation rates for visual acuity testing in 2−3-year-old children, especially among 2-year-olds.

## 1. Introduction

Amblyopia is a neurodevelopmental disorder characterized by impaired development of corrected visual acuity due to abnormal visual input, despite the absence of structural abnormalities in the eye [1]. It is a relatively common visual development abnormality in children. According to recent systematic reviews and meta-analyses, the reported prevalence ranges from 1.36% (95% confidence interval [CI]: 1.27–1.46%) [2], 1.75% (95% CI: 1.62–1.88%) [3], to 1.44% (95% CI: 1.17–1.78%) [4]. Furthermore, the global prevalence of amblyopia is projected to be more than double by 2040 compared to 2019 [4].

Standard treatments for amblyopia include full refractive correction [5] and occlusion therapy [6]. Patching typically requires approximately 2–6 h per day over months [7], and mean adherence is often below 50% [8]. Earlier initiation generally reduces the occlusion time needed and improves outcomes [9,10,11,12]. These considerations underscore the need for early detection—and thus reliable visual acuity assessment—in very young children.

In recent years, various photoscreening devices, such as the Spot Vision Screener, have become widely used for detecting amblyopia. These photoscreeners can objectively and rapidly measure eye position and refractive error; however, they cannot directly assess visual acuity [13]. Moreover, even in cases of moderate to severe hyperopia, amblyopia does not always develop [14,15,16]. Similarly, the presence of strabismus does not necessarily indicate that amblyopia will occur [17,18]. Therefore, when a photoscreener or similar device detects one or more factors suggestive of amblyopia, direct assessment of visual acuity is essential to confirm diagnosis, guide treatment, and enable follow-up evaluation.

The Landolt ring is the international standard for visual acuity charts, as designated by the International Organization for Standardization [19]. However, young children may become bored or fail to understand the optotypes, leading to unreliable, incomplete, or even unevaluable results. To overcome these challenges, pediatric visual acuity charts, such as the Lea Symbols Chart, Allen Figures Chart, HOTV, and the Kay Picture Test, have been developed. Nevertheless, the visual acuity scores obtained from these systems often differ from those measured using standard charts like the Landolt ring or the ETDRS chart [20,21,22,23,24,25]. Furthermore, picture- and symbol-based charts may yield varying visual acuity results, even for optotypes designed to represent the same visual threshold [26]. Although these pediatric charts tend to better capture children’s interest, they generally lack accuracy. For precise assessment, it is important to evaluate minimum separable acuity, which can be reliably measured using the Landolt ring.

**Figure 1:**
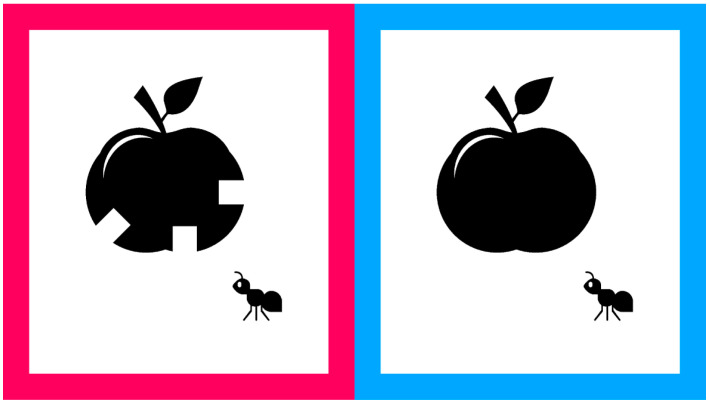
Example of Child-Friendly Minimum-Separable Chart optotypes. The optotype on the left (red border) has gaps similar to those used in the Landolt ring, and it appears to be partially eaten. The optotype on the right (blue border) has no gaps, and it appears not to have been eaten.

**Figure 2:**
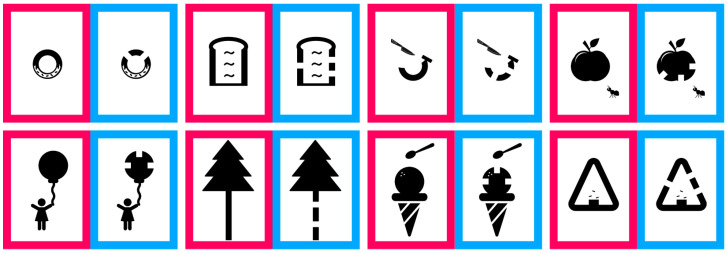
Child-Friendly Minimum-Separable Chart optotypes used in this study.

Therefore, we developed the Child-Friendly Minimum-Separable Chart (CFMS chart), a new visual acuity test designed to maintain children’s interest while ensuring accurate measurement [27]. In the CFMS chart, two familiar images are presented side by side—one on the left and one on the right. One image has portions cut out, while the other remains intact. Visual acuity is assessed by asking the child to identify which image has portions missing. For example, two pictures of an apple are shown: the apple on the left appears partially eaten, while the apple on the right is whole (Figure 1). The examiner asks, “Which apple has been eaten?” and the child responds accordingly. The size of the cut-out portions corresponds to the minimum separable acuity defined by the Landolt ring, allowing the CFMS chart to measure visual acuity equivalent to that of the standard chart [27,28]. Additionally, various optotypes can be created based on this same principle (Figure 2) [27]. Although the CFMS chart has been validated in adults [27,28], its testability in young children has not been evaluated. We hypothesized that the CFMS chart would yield higher cooperation than the Landolt ring in 2–3-year-old children. Therefore, this study aimed to assess the cooperation rates in 2–3-year-old children undergoing visual acuity testing using the Landolt ring and CFMS chart.

## 2. Materials and Methods

A total of 40 children were enrolled, including 20 aged 2 years and 20 aged 3 years. As this was the first study to evaluate the use of the CFMS chart in children, it was designed as a pilot study with a sample size of 20 participants per age group. The mean ± standard deviation of age was 30.6 ± 3.2 months for the 2-year-olds and 41.8 ± 3.9 months for the 3-year-olds. Children who met any of the following exclusion criteria were not included:

- Ocular disorder (other than refractive error) that could affect visual acuity
- Anisometropia with spherical equivalent of ≥2.00 diopters (D)
- Astigmatism > 1.50 D
- Hyperopia > 3.00 D
- Myopia > 1.50 D
- Strabismus
- Refusal to sit in a chair for the test
- Difficulty wearing the trial frame
- Neurodevelopmental disorder, such as developmental delay or autism spectrum disorder

Refractive error was assessed using the Spot Vision Screener, and eye position was evaluated using the cover test.

All study participants underwent visual acuity testing using both the Landolt ring and the CFMS chart. The testing order was randomized using block randomization with a block size of 2, using a computer-generated random sequence with a 1:1 allocation (CFMS-first vs. Landolt-first), and each chart was administered once in a single session (no formal re-test) to minimize order effects. Optotypes were presented on an iPad Pro (4th generation; resolution: 2732 × 2048 pixels; 264 pixels per inch). Ambient room illuminance was maintained at 600–800 l×. The test was performed at a distance of 3 m, chosen a priori to enhance attention and engagement in young children and to improve feasibility in typical pediatric examination rooms. To ensure this distance, the examination chair was fixed at a marked position 3 m from the display, and the examiner monitored the child’s posture and repositioned the child if any deviation from the fixed distance was observed. Only the right eye was tested, with the fellow eye occluded using a trial frame, to minimize examination time and burden in toddlers and to standardize the procedure across participants. Optotypes were displayed in logMAR units, ranging from 1.0 to 0.0 in 0.1-unit increments. Testing began at a visual acuity level that the examiner judged appropriate for the child’s comprehension. The second visual acuity test began at the same visual acuity value used in the first test. All examinations were conducted by a single nationally licensed orthoptist with 10 years of experience in pediatric visual acuity testing, who was independent of the study team and adhered to the study protocol. The primary endpoint was cooperation (testability); inter-ocular differences and binocular function were beyond the scope of this pilot study.

**Figure 3:**
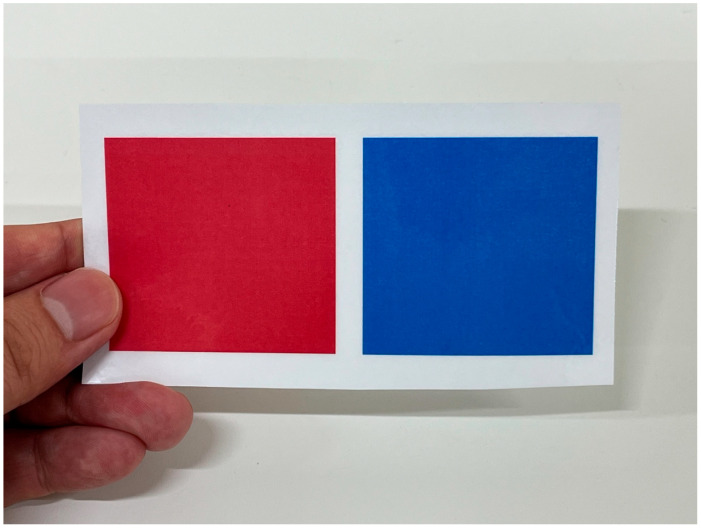
Card with red and blue squares used by the study participants. Participants were instructed to indicate their responses by pointing to either the red or blue section of the card, corresponding to the image on the Child-Friendly Minimum-Separable Chart that contained gaps.

**Figure 4:**
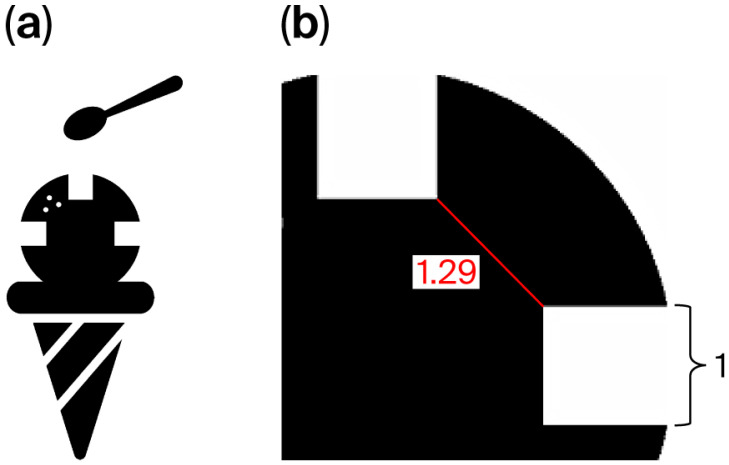
Gaps positioned at the 3 o’clock, 9 o’clock, and 12 o’clock directions in the (**a**) ice cream optotype. (**b**) The minimum distance between adjacent gaps is 1.29 times the length of one gap.

For the visual acuity test using the Landolt ring, participants responded either by pointing or verbally indicating whether the gap was positioned at the top, bottom, left, or right. The optotype was presented as a single letter. According to the Japanese Industrial Standards, when five Landolt rings are shown, a visual acuity level is assigned if the participant correctly identifies at least three (≥60%) [29]. In this setup, the probability of obtaining three or more correct responses by chance is 10.4%. In the CFMS chart, two images were presented side by side, and the participants indicated which image contained the gaps. The image on the left had a red border, while the one on the right had a blue border (Figure 1). Each participant held a response card with a red square on the left and a blue square on the right (Figure 3) and was instructed to point to the red or blue area corresponding to the image with the gaps. Achieving three correct responses out of three presentations was considered sufficient to assign that level of visual acuity (12.5%). This criterion provided a comparable chance accuracy rate to the Landolt ring test, while accounting for the smaller number of optotypes. At each logMAR level, the maximum number of item presentations was fixed by the scoring rule (Landolt ring: five items; CFMS: three items); no additional items or repeats of the same level were allowed. Brief verbal encouragement was permitted but did not increase the number of item presentations. Eight types of optotypes were prepared for the CFMS chart (Figure 2), and participants could choose their preferred designs. If the examiner noticed a loss of interest during testing, the optotypes could be changed to maintain engagement. Each optotype contained multiple gaps placed 90° apart to avoid interference with each other. For instance, in the ice cream optotype, gaps were located at the 3, 9, and 12 o’clock positions (Figure 4a). With a gap length of 1, the shortest distance between two gaps was 1.29 units (Figure 4b). Across all optotypes, the distance between gaps was at least 1.29 times the gap length to prevent overlap and visual confusion.

A pre-test was performed to assess whether participants could understand and cooperate with the visual acuity testing procedure. During the pre-test, logMAR 1.0 equivalent optotypes were presented at a distance of 30 cm, and participants were asked to identify them. The criteria for a correct response were the same as those applied in the main visual acuity test. The pre-test optotypes were printed on paper. If the participant responded correctly to the pre-test, they were deemed capable of understanding and cooperating with visual acuity testing. For the purposes of the cooperation analysis, a child was classified as “cooperative” for a given chart only if the pre-test was passed and at least one logMAR level could be assigned on that chart according to the predefined response criteria (Landolt: 3/5; CFMS: 3/3). Children who did not pass the pre-test or for whom no logMAR level could be assigned were classified as “not cooperative.”

For statistical analysis, the exact McNemar’s test was used to compare cooperation between the Landolt ring and the CFMS chart within each age group (2 and 3 years); this provided paired inference because both charts were administered within subjects. To evaluate the association between age (in months) and cooperation for each chart, binary logistic regression was fitted. In addition to *p*-values, effect sizes for dichotomous cooperation outcomes were reported as absolute risk difference (ARD; CFMS—Landolt), risk ratio (RR), and odds ratio (OR), each with 95% confidence intervals. ARD CIs were computed from binomial proportions (Newcombe/Wilson method), and RR/OR CIs were computed on the log scale. Statistical analyses were performed using BellCurve for Excel, Version 4.06 (Social Survey Research Information Co., Ltd., Tokyo, Japan).

This study was conducted in accordance with the Declaration of Helsinki and was approved by the Japan Orthoptic Vision Society Human Sciences Ethics Committee (Ethics Committee number: 24000072, approval no.: JOVS-25005; approval date: 10 February 2025). The recruitment period was from 11 February to 10 June 2025. All procedures adhered to approved guidelines. Age-appropriate explanations (informed assent) were provided to ensure participants’ understanding, and written informed consent was obtained from parents or guardians.

## 3. Results

**Figure 5:**
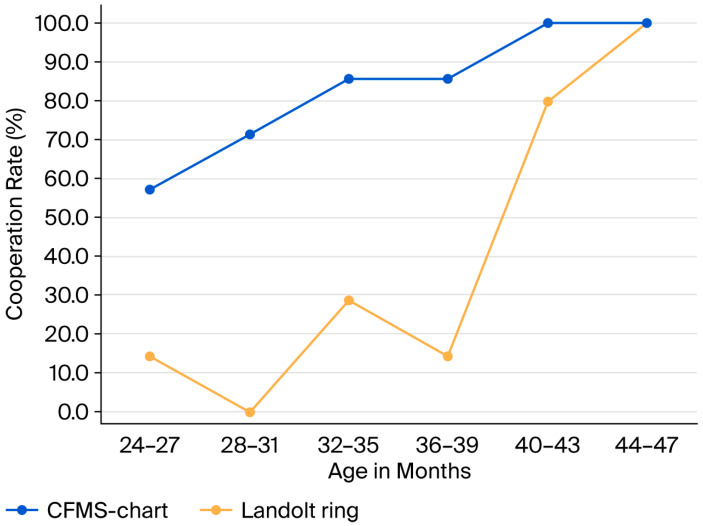
Visual acuity chart cooperation rates for the Landolt Ring and Child-Friendly Minimum-Separable Chart in children aged 24–48 months, stratified by age in 4-month intervals.

Among 2-year-old children, the cooperation rates for the Landolt ring and CFMS chart were 15% and 75%, respectively, indicating a significantly higher cooperation rate with the CFMS chart (*p* = 0.0005) (Table 1). Twelve children who did not cooperate with the Landolt ring successfully cooperated with the CFMS chart, whereas no child showed the opposite pattern. The absolute risk difference (CFMS—Landolt) was +0.60 (95% CI, +0.35 to +0.85); the risk ratio was 5.00 (95% CI, 1.71 to 14.60); and the odds ratio was 17.0 (95% CI, 3.47 to 83.6). Among 3-year-old children, cooperation rates for the Landolt ring and CFMS chart were 60% and 90%, respectively, also showing a significantly higher rate with the CFMS chart (*p* = 0.031) (Table 2). Six children refused the Landolt ring but cooperated with the CFMS chart, and again, no child demonstrated the reverse. Figure 5 illustrates cooperation rates stratified by 4-month age intervals from 24 to 48 months, showing that increasing age was associated with higher cooperation rates. The absolute risk difference (CFMS—Landolt) was +0.30 (95% CI, +0.048 to +0.552); the risk ratio was 1.50 (95% CI, 1.02 to 2.21); and the odds ratio was 6.0 (95% CI, 1.08 to 33.3).

Binary logistic regression analysis assessing the effect of age on cooperation rates revealed that each additional month of age was associated with a significant increase in the odds of cooperation (odds ratio = 1.34, 95% CI: 1.12–1.59, *p* = 0.001). Similarly, for the CFMS chart, each additional month of age resulted in a significant increase in the odds of cooperation (odds ratio = 1.24; 95% CI: 1.03–1.50, *p* = 0.026).

Among the three 2-year-old participants who cooperated with both the Landolt ring and CFMS chart, one participant showed a visual acuity difference of 0.1, while two showed a difference of ≥0.2. In all cases where a difference was observed, the CFMS chart yielded better acuity scores. Similarly, among the twelve 3-year-old participants who cooperated with both the Landolt ring and CFMS chart, six showed no difference in visual acuity, one showed a difference of 0.1, and five showed a difference of ≥0.2. Of these six participants with differing results, one achieved a better score with the Landolt ring, whereas five demonstrated better results with the CFMS chart.

## 4. Discussion

The Landolt ring is the international standard for visual acuity assessment and allows for precise measurement by evaluating minimum separable acuity [19]. However, cooperation rates are low among young children. Previous studies have reported a cooperation rate of 48% in children aged 23−70 months (median: 42.5 months), with particularly low rates among those younger than 30 months [30]. Similarly, the cooperation rate for 3-year-olds was reported to be only 19% when using the FrACT automatic visual acuity measurement device [31]. In the present study, cooperation rates for the Landolt ring were 15% among 2-year-olds and 60% among 3-year-olds, indicating extremely low rates in younger children. Logistic regression analysis further confirmed that cooperation rates significantly increased with age, suggesting that younger children have greater difficulty understanding and engaging with the testing procedure. Beyond statistical significance, the magnitude of improvement was clinically meaningful: among 2-year-olds, cooperation increased by 60 percentage points (ARD 0.60; RR 5.00), corresponding to approximately six additional cooperative children per ten tested; among 3-year-olds, cooperation increased by 30 percentage points (RR 1.50), i.e., about three additional cooperative children per ten tested. These effect sizes support the practical utility of the CFMS chart in 2–3-year-olds.

One likely reason for the difficulty with Landolt ring testing is the requirement to choose from four possible responses per item. In contrast, the CFMS chart requires only two possible responses, making it easier for young children to understand. Instead of identifying whether the gap is on the top, bottom, left, or right, children are simply asked, “Which one has gaps?” A previous study also demonstrated that 3-year-old children showed higher cooperation rates in stereoscopic testing when presented with two-choice questions, indicating that fewer options improve comprehension and cooperation [32]. Several factors likely contributed to the higher cooperation rates observed with the CFMS chart: the simplicity of two-choice questions, the use of engaging images, and the ability of children to choose which pictures to use. In addition, defining success as three correct responses out of three trials likely shortened test duration, helping maintain attention. The CFMS chart also incorporated color discrimination (red and blue) to indicate responses, and since humans can perceive and categorize colors from around 4 months of age [33], this may have further enhanced children’s engagement and cooperation.

Several studies have reported cooperation rates for pediatric visual acuity charts, such as the Lea Symbols and HOTV. One study involving children aged 21−93 months (median: 47 months) reported a 54% cooperation rate for the Lea Symbols, but only 8% among those aged 21−30 months [30]. Another study including children aged 3 months to 3.5 years found cooperation rates of 75% for the Lea symbols and 71% for the HOTV chart [24]. Additionally, cooperation rates of 78% in 3-year-olds and 90% in 4-year-olds have been reported for the Lea Symbols, while HOTV yielded rates of 74% and 88% in the same respective age groups [34]. A separate investigation of the HOTV chart reported cooperation rates of 10% for children aged 24−30 months, 47% for 30−36 months, 80% for 36−42 months, and 93% for 42−48 months [35]. In the present study, the CFMS chart achieved cooperation rates of 75% for 2-year-olds and 90% for 3-year-olds. The difference was particularly notable in the 2-year-old group, with a cooperation rate nearly double that reported for other charts. Both the Lea symbols and HOTV use four-choice formats, similar to the Landolt ring, which likely pose greater difficulty, especially for 2-year-olds [31]. These findings support the CFMS chart’s two-choice design as a more suitable and effective method for assessing visual acuity in this age group. Taken together with prior reports for Lea/HOTV at similar ages [30,31,32,33,34,35], our higher cooperation at age two and meaningful gains at age three—achieved with a two-alternative response format—suggest that reducing cognitive load is a key driver of testability in this age band.

The CFMS chart incorporates various optotypes, allowing children to choose those that match their interests. In theory, the same level of visual acuity should be measured regardless of the chosen optotype [27,28]. However, because these optotypes are more complex than the Landolt ring, the possibility of the crowding phenomenon cannot be excluded. This phenomenon, which peaks in children aged 3−4 years, occurs when nearby shapes make it more difficult for children to identify gaps compared to adults [36]. The potential influence of the crowding effect on the CFMS chart should be examined in future studies.

Unlike the Landolt ring, the optotypes used in the CFMS chart contain multiple gaps. As the Landolt ring is a circular symbol, it is necessary for patients to observe 360° around the circle to identify the gap’s direction. In contrast, the CFMS chart presents two visual symbols side by side. If each symbol had only one gap, children would need to examine both images separately, effectively doubling the visual search effort. Since the CFMS chart is designed specifically for children, the gaps should be as easily detectable as possible; therefore, multiple gaps are incorporated. For instance, in the Landolt ring, when comparing a gap at the 12 o’clock position with one at 3 o’clock, the minimum distance between them is 1.29 times the gap length when the gap length is defined as 1 (Figure 4b). Accordingly, when designing multiple gaps for the CFMS chart, the distance between each was set to be at least 1.29 times the length of a single gap. This spacing ensures that each gap is perceived as an independent visual element, minimizing potential interference even when multiple gaps are present.

In routine pediatric consultations and vision screening, higher testability at ages when amblyopia treatment is most time-sensitive supports earlier VA-based detection and monitoring. The two-choice response format and child-selectable, engaging optotypes may reduce cognitive load, improve cooperation, and shorten test duration, potentially decreasing incomplete examinations and repeat visits. These features could enhance workflow and coverage in busy clinics and community programs; implementation studies are warranted to quantify time savings, training requirements, and cost-effectiveness.

One limitation of this study is that it was conducted as a pilot study at a single facility with a small sample size. As a result, the findings may not be fully representative of the general population, and the precision and external validity of the results are limited. Future validation should involve multicenter studies with larger cohorts. In addition, because participants were recruited from a single outpatient clinic and eligibility required the ability to sit, tolerate a trial frame, and pass a pre-test, selection bias toward relatively cooperative children cannot be excluded, which further limits generalizability. Additionally, visual acuity was measured only in the right eye, with the fellow eye occluded; left-eye acuity and binocular function were not assessed. Accordingly, our findings should be interpreted primarily in terms of testability. Furthermore, because both charts were administered within the same session, learning or fatigue may have influenced performance on the second test; although randomization of test order reduces systematic bias, residual order effects cannot be entirely excluded. The study was not powered to assess between-chart agreement of visual acuity. Thus, our conclusions pertain to testability rather than interchangeability of acuity scores. Owing to the pilot sample size and zero discordant pairs against the CFMS chart, fully adjusted mixed-effects models were considered potentially unstable due to (quasi-)complete separation; thus, we focused on age-stratified within-subject analyses and reported effect sizes with 95% CIs. Although key testing conditions were standardized (room illuminance, fixed viewing distance and chair position, constant display brightness, and a single experienced examiner), the initial starting level was chosen by the examiner rather than fixed a priori; consequently, some residual protocol variability may remain. Future studies will pre-specify starting levels and staircase/stopping rules and incorporate more fully calibrated examination conditions where feasible. Moreover, as the primary objective of this study was to evaluate cooperation rates, test−retest reproducibility and inter-tester reliability could not be assessed. Because the Landolt ring was used as the control, future studies should compare the CFMS chart with other established pediatric visual acuity charts, such as the Lea Symbols and HOTV.

## 5. Conclusions

Among 2–3-year-old children, the CFMS chart achieved higher cooperation than the Landolt ring, with particularly large gains at age two. These findings suggest that a child-friendly, two-choice, minimum-separable design may improve testability in early childhood. However, as a single-center pilot with a small sample, these results should be regarded as preliminary. Larger, multicenter studies—including complete visual-acuity analyses, test–retest and inter-tester reliability, and comparisons with other pediatric charts—are needed to confirm the clinical utility of the CFMS chart for screening programs and routine pediatric practice.

## Figures and Tables

**Table 1:** Cooperation rates for the Landolt Ring and CFMS Chart in 2-year-old children.

2-Year-Old Children	Cooperative with CFMS Chart	Not Cooperative with CFMS Chart	Total
Cooperative with Landolt Ring	3	0	3 (15%)
Not Cooperative with Landolt Ring	12	5	17 (85%)
Total	15 (75%)	5 (25%)	20

CFMS chart, Child-Friendly Minimum-Separable Chart.

**Table 2:** Cooperation rates for the Landolt Ring and CFMS Chart in 3-year-old children.

3-Year-Old Children	Cooperative with CFMS Chart	Not Cooperative with CFMS Chart	Total
Cooperative with Landolt Ring	12	0	12 (60%)
Not Cooperative with Landolt Ring	6	2	8 (40%)
Total	18 (90%)	2 (10%)	20

CFMS chart, Child-Friendly Minimum-Separable Chart.

## Data Availability

The data presented in this study are available on request from the corresponding author. The data are not publicly available due to privacy protection.

## Abbreviations

The following abbreviations are used in this manuscript:

CFMS chart	Child-Friendly Minimum-Separable Chart
CI	Confidence interval
